# In the spotlight: Julio Celis, the Founding Editor of *Molecular Oncology*

**DOI:** 10.1002/1878-0261.13155

**Published:** 2022-01-05

**Authors:** Kevin M. Ryan

**Affiliations:** ^1^ Molecular Oncology FEBS Press Cambridge UK; ^2^ Cancer Research UK Beatson Institute Glasgow UK

1

As we move into 2022, we are not only marking the end of another year, but for *Molecular Oncology*, also a farewell to the journal founder and first Editor‐in‐Chief. The journal was started in 2007 by Julio Celis, who in 2021 decided it was time to step down from the role of Editor‐in‐Chief. Although it is not easy to summarize all of Julio’s achievements as a scientist, policymaker, and founder of the journal, we have sought to hereby present a patchwork of insights from his colleagues and co‐editors, reflecting the impact of his work during all these years.

## 2 From molecular research to science policy

**Fig. 1:**
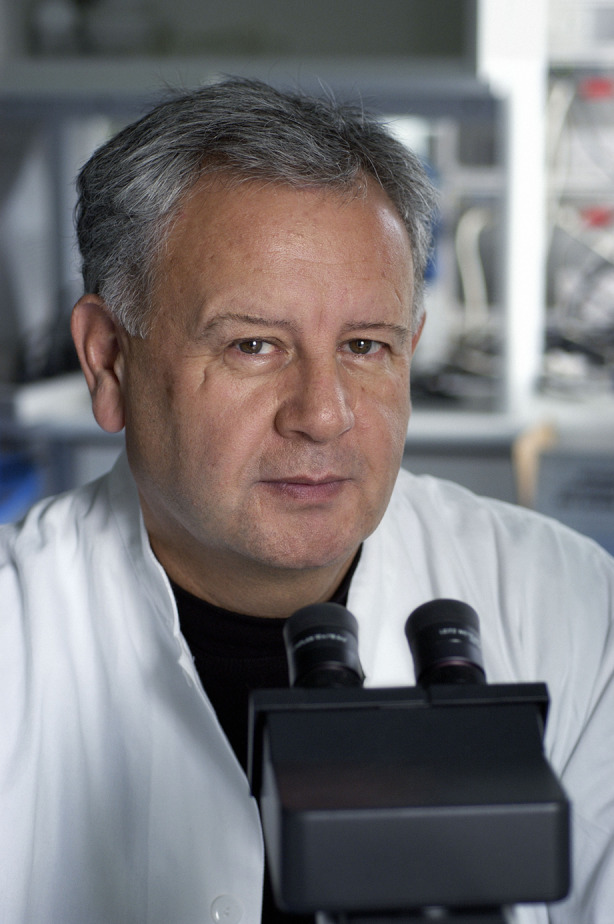
Julio Celis biography. Julio E. Celis conducted post‐doctoral studies at the Medical Research Council Laboratory of Molecular Biology in Cambridge (1971–1973) and was a Member of the Scientific Staff (1973–1975). In 1986, he became professor of Biochemistry at the Institute of Medical Biochemistry at Aarhus University, and later Director of the Institute of Cancer Biology at the Danish Cancer Society until the end of 2011 and Associate Scientific Director of the Danish Cancer Society Research Center until 2021. Prof Celis has been Chairman of the European Molecular Biology Laboratory (EMBL) Council, Vice President of the Human Proteome Organization, President of the Initiative for Science in Europe, President of the European Molecular Biology Conference, Secretary‐General of the Federation of European Biochemical Societies, President of the European Life Sciences Forum, President of the European Association for Cancer Research, Chairman of the Policy Committee of the European Cancer Organisation, Chairman of the Science Policy Committee of the European Academy of Cancer Sciences, Vice‐President of the Alliance for Biomedical Research in Europe, member of the European Commission Research, Innovation and Science Experts (RISE), a High‐Level Advisory Board that advised Commissioner Moedas, Advisor to the Scientific Panel for Health in H2020, Advisor to the board of Cancer Core Europe, and Editor‐in‐Chief of Molecular Oncology. Prof Celis is a Foreign Member of the Royal Danish Academy of Sciences and Letters and a member of the European Molecular Biology Organization, the Academia Europaea, and the European Academy of Cancer Sciences. He is generally recognised as one of the founding fathers of proteomics and played an important role in the steps leading to the creation of the European Research Council. With D. Pavalski, he proposed in 2017 a Cancer Mission for Europe in Horizon Europe.

Julio, originally a native of Chile, moved to the United States for his PhD and to Europe for postdoctoral studies with Sydney Brenner and Francis Crick at the MRC Laboratory of Molecular Biology in Cambridge. He then moved to Denmark as an independent investigator, firstly to Aarhus and subsequently to the Danish Cancer Society in Copenhagen, where he rose to become the Scientific Director of the Institute of Cancer Biology (Fig. 1). As noted by Mef Nilbert, Research Director at the Danish Cancer Society Research Center and Section Editor on *Molecular Oncology*, ‘[Julio,] as a leader of a biological research institute within a major national non‐governmental organization, the Danish Cancer Society, saw the broader impact that basic science has on the patient‐ and population level. He has unique skills in linking molecular cancer biology to outcomes within the clinical fields for patients and their families’. As Ulrik Ringborg, Director of the Cancer Center Karolinska, adds, ‘Julio has been involved for more than 20 years in activities aiming at bridging basic and clinical cancer research. He understood early on the importance of integrating cancer research with cancer therapeutics and care. This ambition to bridge the cancer research with clinical and prevention research aiming at innovations for cancer patients and individuals at risk has been the most stimulating aspect of working with Julio’. Julio’s key activities in the field of science policy, as summarized by Ulrik Ringborg in Box 1, constitute the background for his key contributions to FEBS and the launch and development of *Molecular Oncology*.

## 3 Role in FEBS

Julio served as the Secretary General of FEBS from 1999 to 2007, advocating for equity in research education and funding across Europe. He has been involved in establishing the FEBS Science and Society committee and represented FEBS during the creation of the European Research Council (ERC) in 2007.

Seamus Martin, Professor at Trinity College, Dublin, and Editor‐in‐Chief of *The FEBS Journal*, pays special tribute to the key role Julio played in the process that led to the establishment of the European Research Council (ERC) in 2007: ‘during his tenure as FEBS Secretary General, Julio was central to an initiative to unite the major European life sciences organizations toward the goal of promoting a global vision for life sciences. Following a series of discussions between FEBS (led by Julio in his role as Secretary General) with EMBL (led by Director General, Fotis Kafatos), EMBO (led by Executive Director, Frank Gannon), and ELSO (led by President, Kai Simons), the decision was made to create a European Life Sciences Forum (ELSF). The ELSF was established in 2000 and was headed up by Julio as president of the governing body, which also included Frank Gannon, Fotis Kafatos, and Kai Simons. The ELSF set its course toward supporting scientists in playing a more active role in strategic and science policy issues, increasing the visibility of the life sciences, and, critically, their impact on European policymaking. The ELSF also championed the development of more structured careers for young scientists and the creation of a European Research Council to support basic research.

Following several years of complex discussions with European and National politicians, the ELSF led to the creation of a broader coalition of scientific voices in the shape of the ISE (Initiative for Science in Europe) in 2003. The ISE, initially chaired by José Mariano Gago who was succeeded by Julio in 2005, brought together 35 European scientific organizations to give greater weight to the input of the scientific community on science policymaking in Europe and to promote the ERC initiative. In 2005, the ISE wrote to the Research Ministers of the 25 EU Member States, as well as the European Commission and the Members of the European Parliament, calling for the establishment of an independent European Research Council. Due in large measure to the foregoing efforts, the European Council formally approved the budget for the ERC in 2006, which was officially launched in September 2007. In the years since its inception, the tremendous success of the ERC is a great testament to the vision and tireless efforts of Julio and his ELSF and ISE colleagues, and represents a major landmark in the progression of European scientific research. Julio's skill and passion for the development of large‐scale policy initiatives have given European scientists a strong voice in how science is shaped and supported at a political level, and his dedication and achievements in this sphere have been truly immense’.

## 4 The inception of *Molecular Oncology*

In 2006, Julio proposed the launch of a new oncology journal that would cover all aspects of cancer research, as well as provide a platform for publishing science policy recommendations. Under his guidance, *Molecular Oncology* was initiated as a FEBS Press journal, complementing the existing FEBS journals (*FEBS Letters* and *The FEBS Journal*), and has been published by Elsevier from 2007 to 2016 and Wiley from 2017. As Felix Wieland, Editor‐in‐Chief of *FEBS Letters* at that time, remembers ‘the journal became a great success, owing to Julio’s determination and enthusiasm: in the first years he acted as the sole Editor and set stringent scientific standards that have been kept up with until today’. Julio was assisted by Dorte Holst Pedersen and Jose Moreira, who acted as part‐time Managing Editor. Reacting to the journal’s rapid growth, in 2018 Julio appointed a core team of six Section Editors, to assist with the handling of manuscripts and maintenance of the journal’s quality standards. Since then, the Section Editor team has grown to include fourteen active researchers from across the cancer research continuum.

László Fésüs, who was involved in decisions about *Molecular Oncology* over the past 15 years initially as a member and later chair of the FEBS Publications Committee, recalls long and pragmatic discussions with Julio at meetings, dinners, and often over the phone about the journal’s strategies and development, the transfer from Elsevier to Wiley and transition to open access, and restructuring of the editorial board. ‘I met Julio in the FEBS Council when he was the Secretary General of FEBS and widely recognized both as a renowned pioneer in the proteomics field and as the influential proponent of progressive science policies in Europe which culminated in the foundation of ERC. It was exciting to learn about his determination to launch a new oncology journal with support by FEBS. With Julio’s dedicated efforts, *Molecular Oncology* was supported by many prominent cancer researchers who joined the editorial board. While Julio maintained rigorous editorial standards, the journal reached an IF 6.7 already in 2012. Over the past fourteen years *Molecular Oncology* has become a highly valued asset of the FEBS Press portfolio, while providing a forum for European cancer policy makers and practicing oncologists, which Julio always wanted. A remarkable success story, a highly valuable and laudable achievement’.

Nowadays, *Molecular Oncology* publishes under the gold open access model above 200 papers per year, in all aspects of cancer research — basic, translational, and clinical. In addition, the journal has published some key science policy recommendations, including the 2008 Stockholm Declaration [1], a proposal for a mission‐oriented approach to cancer [2], and the most recent recommendations of European cancer organizations on the cancer mission [3] (Box 1). Maintaining its quality standards, *Molecular Oncology* now has an impact factor of 6.6, and a Citescore of 10. To cite Mef Nilbert, this success may be attributed to the fact that ‘Julio started a scientific journal with a clear scope and vision, and herein also offered a much‐needed space for discussing science policy. Through his work combining science and policy, *Molecular Oncology* is a common ground to make a positive impact, which is especially timely and relevant in the current work with the CEU Beating Cancer Plan and the Cancer Mission’.

Box 1 **The contributions of Julio Celis to European Cancer Policy, as documented by Ulrik Ringborg.**

1 At the beginning of the century, the EU Commissioner for Research and Education Philippe Busquin pointed out the necessity to address cancer research fragmentation. Julio Celis was then member of an advisory group working on the planning and implementation of the EUROCAN+Plus 2005‐2007 project, which resulted in some key recommendations on the need for establishing Comprehensive Cancer Centres to integrate research with healthcare, and for promoting institutional collaborations to ensure research sustainability and critical mass, both required for the development of precision/personalized cancer medicine. The resulting Stockholm Declaration was signed by representatives from 17 cancer research centers, setting the grounds for a European institutional translational cancer research collaboration [1].

In this spirit, Julio suggested the establishment of the European Academy of Cancer Sciences (EACS) at a Cancer Policy meeting in Paris. After the EACS was founded in 2009, Julio organized a meeting with scientists and policy and decision‐makers at the UNESCO building in Paris to prepare for the next step: to convince the European Commission to allocate economic support for the analysis of collaboration among institutes for translational cancer research. The EurocanPlatform project was thus initiated in 2011, and under Julio’s recommendation, EACS would provide advice on the sustainability of potential deliverables. The EurocanPlatform project led to the creation of Cancer Core Europe (a consortium of seven cancer research centers) for therapeutic research and Cancer Prevention Europe (a consortium of ten cancer research centers) for prevention research.

As the chair of the Science Policy Committee of EACS, which was created with the accession to EACS of the ECCO Cancer Policy Committee, Julio had extensive contacts in the European Commission and the European Parliament, including Mariano Gago, who recommended Julio as an advisor to the new Commissioner for Research and Innovation, Carlos Moedas. Julio also represented the Life Sciences at the RISE High‐Level Group, and presented to Carlos Moedas a mission‐oriented approach to cancer based on evidence documented by EACS. Julio’s systematic work helped to further develop specific policy recommendations to the Directorate‐General for Health and Food Safety (DG Sanco) and Directorate‐General for Research and Innovation (DG RTD) of the European Commission. He involved Commissioners and research ministers in policy conferences, including the two Gago conferences on European Science Policy and a conference arranged together with the Pontifical Academy of Sciences in the Vatican 2018. Following these events, the European Commission decided to finance a Mission on Cancer. Together with the EACS President, Anton Berns, Julio coordinated the publication of some recommendations shared by main European cancer organizations about the strategies for cancer research and infrastructures [3].

In parallel, Julio supported Miklós Kásler, former head of the National Institute of Oncology Budapest and Minister of Human Resources in Hungary, in creating the Central Eastern European Academy of Oncology, now connecting 21 countries for international collaboration with the aim to decrease present inequalities. He also contributed to the development of the accreditation methodology by the Organisation of European Cancer Institutes, and launched in 2012 a successful series of annual summer school events devoted to translational cancer research and policy, now continued under responsibility of the Cancer Core Europe.

## 5 Colleagues share their thoughts about Julio’s contributions

**Heike Allgayer**, Professor at the University of Heidelberg and *Molecular Oncology* Section Editor: ‘Although I already knew Julio’s prominent role in translational research and European science policy, I was still impressed by his active enthusiasm and vision when I first met him personally at an EACS meeting, where we had inspiring discussions about the future of European Cancer Research. Shortly after, I enthusiastically accepted his invitation to act as a Section Editor for the Metastasis and Translational field in *Molecular Oncology*. Since then, I have experienced Julio not only as an inspiring visionary, but also as an incredibly supportive colleague and mentor. I sincerely wish him all the best for his future, which, as I am sure, will still be filled with many creative ideas and initiatives for cancer research across Europe and beyond’.

**Anton Berns**, EACS President and *Molecular Oncology* Editorial Board Member: ‘As its Editor‐in‐Chief, Julio Celis has made *Molecular Oncology* a very respectable journal that publishes excellent basic and translational cancer research. Importantly, under his guidance, *Molecular Oncology* has developed to become an accessible and highly appreciated platform to update the scientific community on science policy issues. Julio’s tireless efforts to inform European policymakers of the need to stimulate cancer research have significantly contributed to the launch by the EU commission of the EU Mission on Cancer and Europe's Beating Cancer Plan. Furthermore, as chairman of the policy committee of the European Academy of Cancer Sciences he has immensely served the scientific community and the society at large by articulating what is needed for effective implementation of these initiatives, all to the benefit of cancer patients. Importantly, all these recommendations have been disseminated through their publication in *Molecular Oncology*. We owe him a debt of gratitude’.

**Michael Boutros**, German Cancer Research Center (DKFZ), Professor at Heidelberg University and *Molecular Oncology* Section Editor: ‘Julio has contributed tremendously to European science and science policy and has a lasting impact on connecting basic, translational and clinical cancer research and on the new Cancer Mission of the European Commission. It was a great honor serving with him on the Editorial Board of *Molecular Oncology*.’

**George Calin**, Professor of Translational Molecular Pathology and Leukemia at the MD Anderson Cancer Center in Houston, Texas, and *Molecular Oncology* Section Editor: ‘Although I had never met Julio in person, I was immediately attracted by his proposal to become a Section Editor on *Molecular Oncology* because I knew well his tremendous achievements as a scientist (his pioneering work on cancer proteomics), as a science policy maker (I believe creation of ERC is a fantastic achievement for European science), and as a founding editor of an oncology journal publishing both basic research and translational findings. It was Julio’s personality that convinced me to add a new role on a quite full plate. And I have never regretted that decision, as working with Julio and the excellent team supporting the journal, was a day‐by‐day learning experience for me on how to build a successful journal from scratch. I enjoy and appreciate Julio’s balanced attitude in supporting the new initiatives needed for the journal's development in the very competitive field of molecular oncology, Julio’s kind and calm way to discuss and approach problems and, generally, his positive attitude that glued together the members of a successful team. I wish him success in everything he is doing further and I am thankful that I learnt from him so much on how to build and lead a successful scientific journal. This is a very precious mentoring, as science is mostly about publishing the discoveries!’

**Miguel A. De la Rosa**, Professor at University of Seville and Editor‐in‐Chief of *FEBS Open Bio*: I had the opportunity to first appreciate Julio’s leadership and foresight at the beginning of the 2000s, when he was FEBS Secretary General. The key role he played at that time in promoting the movement leading to the creation of the ERC was a premonition of his successful launching of *Molecular Oncology* soon after.

**Seamus Martin**, Professor at Trinity College, Dublin, and Editor‐in‐Chief of *The FEBS Journal*: ‘I have had the privilege to get to know Julio during the annual meetings of the FEBS Publications Committee, and to benefit from his immense scientific, leadership and political experience, not to mention his good humor and ready smile. There are few scientists that have made such sustained and influential contributions to European research, as well as science policy, on the scale that Julio Celis has. It is clear that Julio's dedication to the development of high‐level initiatives to foster the development of European cancer research, as well as scientific research in its broadest sense, still burns with an intensity that is highly unusual.

On a personal level, Julio is a true gentleman and it has been a pleasure and honor to work with him, alongside other FEBS Press editors, to contribute to the development of the growing portfolio of FEBS journals, including *Molecular Oncology*. His legacy as founding Editor‐in‐Chief of *Molecular Oncology*, as well as a key figure in the shaping of European scientific institutions and science policy is secure’.

**Mef Nilbert**, Research Director at the Danish Cancer Society Research Center, and *Molecular Oncology* Section Editor: ‘Julio’s ability to bring science to the table of EU commissioners and decision‐makers stirs hope for the future and inspires a new generation of basic researchers to consider clinical value. Julio has led a common ground for future positive impact'.

**Vaclav Paces**, FEBS Secretary General: ‘While serving as FEBS Secretary General, I have appreciated how much FEBS owes to Julio. I particularly value Julio’s contribution toward supporting research in Central and Eastern Europe, already in the 1990s. When Julio was FEBS Secretary General, he contributed to the program of Peter Campbell by sending laboratory equipment to Czechoslovakia and other Central and Eastern European Countries. Julio also invited Czech young scientists to his laboratory and gave them full support. As highlighted in my article entitled 'Biochemistry Behind the Iron Curtain’, which was part of the book published at the occasion of 50 years of FEBS existence, I have appreciated these efforts of FEBS, under Julio’s guidance, to integrate colleagues formerly working behind that Iron Curtain’.

**Klaus Pantel**, Director of the Institute of Tumor Biology, University Medical Center Hamburg‐Eppendorf and *Molecular Oncology* Section Editor: ’I would like to thank Julio very much for his enormous work for our journal. His wonderful enthusiasm has “infected” all of us and the quality of the journal has largely profited from his never ending energy to screen manuscripts and provide guidance. Besides Julio has been a great supporter of European Research and contributed to the establishment of the EU as [an] active and successful research community. It is hard to imagine an Editorial Board meeting without Julio and I wish him all the best for his future.’

**Ulrik Ringborg**, Director of the Cancer Center Karolinska and *Molecular Oncology* Senior Editor: ‘The European Mission on Cancer and most probably also the Europe´s Beating Cancer Plan might have never materialized without Julio’s systematic work and strategical thinking about new types of research collaborations and infrastructures toward precision and personalized medicine in cancer. I can still remember that during one of my first visits to Julio in Copenhagen, and while discussing his own research, Julio had already focused on observations that had the chance to improve diagnostics and treatment of patients. He saw the strong potential of cancer biology to improve the lives of cancer patients, and was aware of the obstacles in clinical and prevention research. In the many discussions that followed over the last two decades, Julio, often iterated his view that cancer research is there for the patients. This concept has now been integrated in European policy, in terms of support to translational research covering a complete cancer research continuum for all components of cancer treatment, care and prevention’.

**Felix Wieland**, Professor at University of Heidelberg and former Editor‐in‐Chief of *FEBS Letters*: ‘While serving as Secretary General of FEBS and later as a member of the Publication Committee, Julio has contributed enormously to our Society and to the FEBS Press publications’.

## 6 Looking ahead

I joined *Molecular Oncology* as co‐Editor‐in‐Chief alongside Julio, at the beginning of 2020. During our time working together, I learned a great deal from Julio about the journal and publishing in general. As I have now taken the role of sole Editor‐in‐Chief, I hope that together with the Editorial Office, Section Editors and Editorial Advisory Board we can continue the success and growth of *Molecular Oncology*. While we mark and give thanks to all of Julio’s dedication to the journal, it is not a goodbye, as Julio remains in open communication with the journal. Julio also continues with his many endeavors with the European Academy of Cancer Sciences and the European Commission. We wish him every success and look forward to reporting further significant developments of these efforts in *Molecular Oncology*!
